# Proteomic analysis reveals a biosignature of decreased synaptic protein in cerebrospinal fluid of major depressive disorder

**DOI:** 10.1038/s41398-020-0825-7

**Published:** 2020-05-12

**Authors:** MHD Rami Al Shweiki, Patrick Oeckl, Petra Steinacker, Peggy Barschke, Cornelia Dorner-Ciossek, Bastian Hengerer, Carlos Schönfeldt-Lecuona, Markus Otto

**Affiliations:** 1 Department of Neurology, Ulm University, Ulm, Germany; 2 CNS Diseases Research, Boehringer Ingelheim Pharma GmbH & Co. KG, Biberach an der Riss, Germany; 3 Department of Psychiatry and Psychotherapy III, University of Ulm, Ulm, Germany

**Keywords:** Biomarkers, Neuroscience, Pathogenesis

## Abstract

Major depressive disorder (MDD) is a leading cause of morbidity with a lifetime prevalence of 10%. There is increasing evidence suggesting synaptic dysfunction and impaired integrity of certain brain circuits in MDD. Here we investigate the cerebrospinal fluid proteome of psychiatric patients focusing on MDD by deep proteomic profiling approach combined with a further validation step using targeted mass spectrometry. We demonstrate profound CSF proteomic changes during on-going depression episodes in MDD patients (*n* = 40) in comparison to controls (*n* = 27), schizophrenia spectrum disorder (*n* = 13), and bipolar disorder patients (*n* = 11). The discovery analysis with isobaric tags for relative and absolute quantitation (iTRAQ) reveals changes in proteins associated with synaptic transmission, myelination, and Wnt signaling in CSF of MDD. The multiple reaction monitoring (MRM) validation analysis confirms significantly decreased levels of eight proteins including the membrane synaptic proteins neurexin 3 (NRXN3), contactin-associated protein-like 4 (CNTNAP4), and glutamate ionotropic receptor AMPA type subunit 4 (GRIA4) in the CSF of MDD patients in comparison to the controls. Overall, the study demonstrates proteins that constitute an MDD biosignature for further validation studies and provides insight into the pathophysiology of MDD and other psychiatric disorders.

## Introduction

Major depressive disorder (MDD) is a leading cause of morbidity, severely affecting the productivity of society. It has a lifetime prevalence of 10%^1^ and its economic burden is approximately 92 billion euros per year in Europe^2^. MDD is a debilitating disorder with a negative impact on personal and family life^3^ and considered as a risk factor for devastating disorders, such as Alzheimer’s dementia^4^ and cardiovascular disease^5^. MDD is a mood disorder particularly affecting emotional functioning with recurring episodes of increasing severity and resistance to antidepressants across episodes^6^. In treatment-resistant MDD patients, attempted or actual suicide is of high incidence^7^.

MDD is highly heterogeneous in terms of etiology, symptom presentation, course, and response to treatment. Several hypotheses have been proposed for explaining the pathophysiology of MDD, including altered neuro-circuits activity, monoamine deficiency, neurotrophic alterations, glucocorticoid dysregulation, immuno-inflammation, imbalance in the microbiome, disturbed energy metabolism, and oxidative stress^8–11^. However, the current understanding of MDD pathophysiology is still incomplete.

Despite the extensive research on MDD genetics, no single genetic locus has strongly been associated with an increased risk of MDD. Multiple genetic factors together with environmental factors may play a role in the development of MDD^12^. In contrast to genetics and transcriptomics, proteomics measures thousands of proteins that represent the major functional molecules in a biological system, and hence, proteomic profiling provides a closer approximation to the dynamic pathophysiological processes. Interestingly, a recent proteomic analysis of postmortem MDD patients’ brains revealed changes in synaptic proteins that were consistent across episodes and remission phases suggesting a persistent molecular MDD pathology^13^. Given the complex nature of MDD, proteomics has a great potential to improve our understanding of MDD pathophysiology and to identify MDD biosignatures.

Cerebrospinal fluid (CSF) presents a rich source of biosignatures for neuropsychiatric disorders due to its direct contact with the brain^14,15^. For several neurological disorders (e.g., amyotrophic lateral sclerosis and Alzheimer’s disease), researchers were able to identify disease biomarkers in CSF^16,17^. However, merely a small number of studies investigated the CSF proteome of MDD patients with a low proteomic coverage^18,19^.

The aim of our study was to investigate the proteomic changes in the CSF of psychiatric patients focusing on MDD by the deep proteomic profiling isobaric tag for relative and absolute quantitation (iTRAQ) approach followed by a further validation step using the high-powered targeted mass spectrometry (MS) multiple reaction monitoring (MRM) approach. We describe the proteomic changes in CSF during on-going depression episode in medicated MDD patients in comparison to controls as well as other mental illnesses (schizophrenia (SCZ) spectrum and bipolar (BI) disorder). We validate our discovery iTRAQ findings by measuring the CSF levels of 12 brain-enriched proteins that showed differential expression in the discovery analysis in a relatively large cohort of psychiatric patients using MRM. The study provides insight into the pathophysiology of psychiatric disorders, mainly MDD, and demonstrates biosignatures.

## Materials and methods

### Chemicals and reagents

iTRAQ multiplex kits (4-plex) (#4352135) were obtained from Applied Biosystems (AbSciex Inc., USA). Liquid chromatography–MS (LC-MS)-grade ethanol, methanol, acetonitrile, and water were purchased from Carl Roth GmbH + Co. KG (Karlsruhe, Germany). LC-MS-grade formic acid, trifluoroacetic acid (TFA), and Tris (2-carboxyethyl) phosphine hydrochloride (TCEP) were from Thermo Fisher Scientific GmbH (Dreieich, Germany). Trypsin/Lys-C mix was purchased from Promega GmbH. Solid-phase extraction disks were obtained from Empore SCX 2240 3M. Chloroacetamide (CAA) (C0267-100G) was purchased from Sigma (Steinheim, Germany). Ammonium hydroxide solution (25%) was from Merck (Darmstadt, Germany), and triethylammonium bicarbonate (TEAB) was from Fluka (Seelze, Germany). Flagellin (FPB3801) was purchased from Invivogen (CA, US); β-lactoglobulin (1511098) was from Thermo. Neurexin 3 antibody (AF5269) was from R&D Systems (Minneapolis, USA). Synthetic heavy peptides of cerebellin-4 (CBLN4) [36–49, 51–55] and proprotein convertase subtilisin/kexin type 1 inhibitor (PCSK1N) [199–218] were obtained from JPT Peptide Technologies GmbH (Berlin, Germany). VGF QPrEST peptide sequence was purchased from Atlas antibodies (Bromma, Sweden). Cocaine- and amphetamine-regulated transcript protein (CARTPT) [73–93], CARTPT [102–116], carnosine dipeptidase 1 (CNDP1) [242–256], CNDP1 [74–89], contactin-associated protein-like 4 (CNTNAP4) [884–900], glutamate ionotropic receptor AMPA type subunit 4 (GRIA4) [215–232], leucine-rich repeat and immunoglobulin-like domain-containing nogo receptor-interacting protein 1 (LINGO1) [59–76], LINGO1 [389–407], neuronal pentraxin receptor (NPTXR) [234–253], NPTXR [277–297], NRXN3 [1240–1257], neurexophilin 1 (NXPH1) [55–66], neuroserpin (SERPINI1) [257–274], and SERPINI1 [285–296] were purchased from Thermo Fisher Scientific (Munich, Germany). The detailed peptide sequences and protein names are provided in Supplementary Table 1.

### Patients and CSF collection

The study was approved by the Ethics Committee of Ulm University (No.20/10). Each patient provided written consent to participate in the study, which was conducted according to the institutional guidelines. Psychiatric patients were enrolled at the Ulm University Hospital and characterized according to Diagnostic and Statistical Manual of Mental Disorders, Fifth Edition criteria^20^. Patients of the control group (Con) were screened with a semi-structured interview to exclude psychiatric disorders. The controls did not show any inflammatory or neurodegenerative process in CSF analysis as they showed normal cell count, lactate, quotient of immunoglobulin G (IgG), quotient of albumin, oligo-clonal IgG bands, and neurofilament light chain levels. They did not receive any psychiatric medication. In addition, they showed no elevation in the levels of serum neurofilament light chain^21^ and normal cranial magnetic resonance imaging scans. The diagnoses of the non-psychiatric controls were as follows: numb feeling in the hand (1), benign paroxysmal positional vertigo (3), migraine (5), anterior ischemic optic neuropathy, diabetes mellitus (DM), arterial hypertension (1), tension headache (2), retinal ischemia right eye, DM (1), nonsystematic vertigo, arterial hypertension (1), radiculopathy, spinal stenosis (3), eyelid ptosis with swelling of the eyelid may be due to allergic reason, hypothyroidism (1), foreign body in the conjunctival sac (1), chronic fatigue syndrome (1), idiopathic vasovagal presyncope (1), labyrinthitis (2), Morbus menière (1), neurological check to exclude a suspicion of cerebral ischemia (1), artery occlusion A. temporalis superior—eye thrombosis (1) and liability to Pressure Palsies (Plexus brachialis) (1). A detailed description of the controls with CSF neurofilament light values is provided in Supplementary Table 2. CSF was collected by a lumbar puncture; samples were centrifuged and stored at −80 °C for further analysis. The discovery iTRAQ cohort consisted of 38 subjects: 12 MDD patients (10 recurrent depressive episode International Classification of Diseases (ICD)-F33 and 2 depression episode ICD-F32), 6 BI patients (3 mixed ICD-F31.6, 2 manic ICD-F31.1, and 1 depressive episode ICD-F31.3-5), 6 SCZ patients (4 paranoid ICD-F20.0 and 2 undifferentiated ICD-F20.3), and 14 controls. For the validation of our identified proteomic changes, we enlarged our cohort to comprise 91 subjects: 40 MDD patients (30 recurrent depressive episode ICD-F33 and 10 depression episode ICD-F32), 11 BI patients (4 mixed ICD-F31.6, 3 manic ICD-F31.1, and 4 depressive episode ICD-F31.3-5), 13 SCZ patients (7 Paranoid ICD-F20.0 and 6 undifferentiated ICD-F20.3), and 27 controls. Depression severity was assessed using the Montgomery–Asberg Depression Scale (MADRS). The medications of the MDD, SCZ, and BI patients taken at CSF collection time are provided in Supplementary Table 3. The sample sizes were determined by samples’ availability.

### Sample preparation for discovery LC-MS analysis

After spiking of each 200 µL CSF sample with 40 µL internal standard solution (200 ng Flagellin 10 ng Ovalbumin, and 2.5 pmol β-Lactoglobulin), samples were reduced and alkylated with 5 mM TCEP and 10 mM CAA for 30 min, 60 °C, 400 rpm, respectively. Samples underwent a buffer exchange with 500 mM TEAB four times using Microcon 3-kDa centrifugal filters. The concentrated protein extracts were digested for 16 h at 27 °C and 600 rpm with trypsin/lysine-C mix at 50:1 protein-to-enzyme ratio. The digested peptides were mixed with 75 µL 75% ethanol and subsequently labeled with iTRAQ labels. The samples were concentrated overnight in a speed vacuum and fractionated by in-house prepared strong cation exchange STAGE Tips. Peptides were eluted using 125 mM ammonium acetate/20% acetonitrile/0.5% formic acid (fraction 1), 160 mM ammonium acetate/20% acetonitrile/0.5% formic acid (fraction 2), 225 mM ammonium acetate/20% acetonitrile/0.5% formic acid (fraction 3), 300 mM ammonium acetate/20% acetonitrile/0.5% formic acid (fraction 4), 450 mM ammonium acetate/20% acetonitrile/0.5% formic acid (fraction 5), and 5% ammonium hydroxide/80% acetonitrile (fraction 6). The fractions were evaporated in a speed vacuum and re-dissolved in 12 µL 0.5% TFA. The peptide concentration was determined by absorption at 280 nm. The CSF samples were analyzed in a random sequence.

### Sample preparation for MRM analysis

After spiking of each 200 µL CSF sample with the diluted heavy synthetic peptide standards, the samples were reduced and alkylated with TCEP and CAA, respectively. Then the samples were digested for 16 h with 1 µg of trypsin/lysine-C mixture. The digestion was stopped by adding 10 µL of a 35% TFA solution. The fractionation was performed as in the discovery approach. The fractions were evaporated in a speed vacuum, re-dissolved in 27.5 µL 0.5% TFA/6% ACN, and 20 µL from each fraction were injected into the high-performance LC (HPLC)-MS system. The 91 CSF samples were analyzed in two runs, the first run included 17 MDD, 5 BI, 6 SCZ, and 14 Con and the second run included the rest of the samples. The two runs were subsequently measured on the mass spectrometer, with a break of 2 days in between (a weekend). In each run, we measured a CSF pool five times across the run (at the beginning and after every 10–12 samples to the end of the run) to monitor for the stability. The average of these five replicates was used to determine the inter-assay variation and to normalize for any batch effect. The CSF samples of the patients and controls were analyzed in a random sequence in the mass spectrometer. To determine the intra-assay variation of the measured peptides, four technical replicates of a control CSF sample were measured in a row on the mass spectrometer.

### Discovery LC-MS analysis

The samples were injected into an Ultimate 3000 RSLC nano-system (Thermo Fischer Scientific). Peptides were trapped using an Acclaim PepMap 100 pre-column (200 × 0.075 mm, 3 μm) and then separated using an Acclaim PepMap RSLC analytical column (500 × 0.05 mm, 2 µm); both columns were purchased from Thermo Fisher Scientific. The peptides were eluted using mobile phase A (4% dimethyl sulfoxide (DMSO) in 0.1% formic acid) and B (76% acetonitrile, 4% DMSO in 0.1% formic acid) with a 360-min multistep gradient. For fractions 1 and 2, the gradient was increasing from 1% B to 20% B in the run time between 5 and 220 min, followed by increasing from 20% B to 53% B in the run time between 220 and 310 min. For fractions 3 and 4, the gradient was increasing from 1% B to 32% B in the run time between 5 and 220 min, followed by increasing from 32% B to 53% B in the run time between 220 and 310 min. For fractions 5 and 6, the first step included increasing B concentration from 1% to 10% from 5 to 40 min, followed by an increasing gradient from 10% B to 32% B from 40 to 240 min and later from 32% B to 53% B from 240 to 310 min. For all fractions, the column was washed with 99% B and equilibrated to start conditions between 310 and 360 min of the run time. Upon elution, the peptides were electro-sprayed into a Q-Exactive mass spectrometer (Thermo Fischer Scientific). The source voltage was set to 2.3 kV and 270 °C. Data were acquired by data-dependent acquisition (Top12) with the following settings: full MS: resolution 70,000, AGC target 3e6, max injection time 120 ms, scan range 400–1400 *m*/*z*, MS^2^: resolution 35,000, AGC target 1e6, max injection time 120 ms, isolation window 1.6 *m*/*z*, NCE 25, fixed first mass of 100 *m*/*z*, and a dynamic exclusion of 40 s.

### Targeted LC-MS analysis

The MRM analysis was carried out using an Agilent 1260 HPLC pump (Santa Clara, CA), Eksigent microLC200 (AB Sciex, Framingham, MA), and AB Sciex QTRAP6500 mass spectrometer in a positive ionization mode (AB Sciex, Framingham, MA). Twenty microliters of sample were loaded from a cooled auto-sampler (4 °C) on a C18 PepMap100 (5 × 0.3 mm, 5 μm) trap column (Thermo Fisher Scientific) using mobile phases A and B, which consisted of 0.05% TFA and 0.05% TFA in MeOH, respectively. The multistep gradient is described in Supplementary Table 4. Afterward, peptides were separated on an Eksigent HALO Fused-core C18 (100 × 0.5 mm, 2.7 μm) column at 40 °C with mobile phases A and B, which consisted of 4% DMSO/0.1% formic acid and 4% DMSO/96% ACN/0.1% formic acid, respectively (Supplementary Table 4). The analytical column was connected to the QTRAP6500 with a 25 µm electrode, and the instrument was set to a scheduled MRM mode (retention time window 90 s, scan time 1 s). The ion source settings were set to 5500 V, 175 °C, curtain gas (CUR) 40 psi, nebulizer gas (GS1) 40 psi, GS2 30 psi, and CAD gas high. The analyzed transitions and implemented MS settings are described in Supplementary Table 5.

### Immunoblotting

Identical volumes of 22 μL of native CSF were mixed with sodium dodecyl sulfate-polyacrylamide gel electrophoresis sample buffer (Roti-load 1; Carl Roth GmbH, Karlsruhe, Germany) to a final concentration of 2.5% mercaptoethanol and cooked for 5 min at 95 °C. The samples were loaded on 4% acrylamide stacking gel. Proteins were separated on 8% acrylamide separation gel at 25 mA per gel for about 90 min. Proteins were transferred to polyvinylidene difluoride membranes (Millipore Corporation, Bedford, MA) by semidry blot. Membranes were blocked with 5% dry milk in phosphate-buffered saline and 0.075% polysorbate 20 (Tween-20) (Bio-Rad, Hercules, CA). After blocking, membranes were incubated with Neurexin 3 antibody (1:1000) in the blocking buffer overnight at 4 °C. After 3 washing steps, the membranes were incubated for 1 h at room temperature with peroxidase-conjugated rabbit anti-sheep (DAKO, Glostrup, Denmark) secondary antibody. A western blot detection reagent (ECL Plus; GE Healthcare) was used as a substrate, and chemiluminescence was measured with a charge-coupled device camera (LAS-1000; Fujifilm, Tokyo, Japan).

### Data analysis

Protein identification and quantification were performed using MaxQuant 1.5.2.8^22^. For identification, *Homo sapiens* reference proteome from UniProt (downloaded 04-Feb-2017) was used as a reference database. The enzyme specificity was set to trypsin excluding cleavages before proline, and two missed cleavage sites were tolerated. Carbamidomethylation of cysteine and acetylation of terminal nitrogen were set as fixed modifications. Oxidation of methionine was set as a variable modification. For quantification, the reporter ion MS2 intensities of 4 plex iTRAQ with a minimum precursor ion fraction of 0.75 were adopted. A false discovery rate (FDR) of 1% was used for peptide and protein identification. The data analysis was conducted with Perseus 1.6.6.0.^23^. Contaminant proteins and proteins identified with <2 unique peptides were excluded. First, the measured samples in different multiplexes were normalized to a CSF pool that was labeled with 114 iTRAQ reagent in each multiplex, then the data was normalized to the spiked internal standards. A dataset of 920 proteins that were quantified in at least 65% of the samples in each group (i.e., 9 controls, 4 SCZ, 4 BI, and 8 MDD) was used in the data analysis when all groups were included. The non-supervised hierarchical clustering analysis was performed using Pearson correlation after normalization to *z*-score. Student’s *t* test with permutation-based FDR of 0.05 was conducted to identify differentially regulated proteins. Student’s *t* test was applied to the dataset that included at least 65% of the samples in each group of the respective comparison. PerseusAnnot.txt.gz (http://141.61.102.106:8080/share.cgi?ssid=0qF9uFn#0qF9uFn/FrequentlyUsed,mainAnnot.homosapiens, 2015) file was used to provide gene ontology (GO) biological process (BP), molecular function, and cellular component annotation to the data. Fisher exact test and Benjamini–Hochberg FDR of 0.05 were performed to determine the enriched GO terms. Similarity to CSF albumin levels (albumin profile) was estimated using Spearman distance.

For the targeted data, the raw data files were imported to Skyline, peak picking was carefully controlled, and the ratio of light to heavy peptide intensity was used as readout. To overcome any batch effect, the data were normalized to a CSF pool that was measured five times in all batches. The statistical analysis was performed using GraphPad Prism 5.0. Disease groups were compared by Kruskal–Wallis test and Dunn’s Multiple Comparison Test. Correlation analysis was performed using Spearman’s correlation coefficient. Two-way analysis of variance (ANOVA) was used to study the effect of gender on the MDD and control data; after testing for normal distribution, the disease was set as a first variable and the gender was set as a second variable.

Densitometric analysis of immunoblots was performed using the ImageQuant-TL software, neurexin 3 (NRXN3) expression was normalized to a CSF pool that was loaded to all blots, and the normalized intensities were compared by Student’s *t* test (two tailed).

## Results

### Clinical characterization

Demographic characteristics, percentage of suicide attempters, and percentage of patients with elevated CSF/serum albumin ratio for MDD, BI, and SCZ patients and Con are summarized in Table 1. The MDD, BI, SCZ, and control groups did not differ concerning age in the discovery iTRAQ (*p* = 0.1559) and MRM (*p* = 0.0932) cohorts. The gender distribution in the MDD group including more females than male participants was comparable to the control group in the discovery iTRAQ and MRM cohorts. The patients’ groups (MDD, SCZ, and BI) did not differ in body mass index (BMI) in the discovery iTRAQ (*p* = 0.611) and MRM (*p* = 0.670) cohorts. The studied MDD patients and controls demonstrated a normal blood–CSF barrier function reflected by CSF/serum albumin ratios laying within the reference ranges of the different age categories^24^. In the MRM cohort, 38% of the studied SCZ patients and 45% of the studied BI patients showed elevated CSF/serum albumin ratio (Table 1).

**Table 1:** Clinical characterization of the study cohorts.

Characteristic	MDD	BI	SCZ	CON	*p* Value
Discovery iTRAQ cohort					
Group size	12	6	6	14	–
Subgroups	Recurrent depressive disorder (10)	Current episode mixed (3)	Paranoid (4)	–	–
		Current episode manic (2)			
	Depressive episode (2)	Current episode depression (1)	Undifferentiated (2)		
Age	46 ± 13.3	47.7 ± 11.4	36.3 ± 12.7	50.2 ± 9.8	0.1599
	(19.9–69.2)	(33–58)	(23.8–56.7)	(29.4–65.6)	
Sex F/M	10/2	3/3	1/5	10/4	–
Female	83.3%	50.0%	16.7%	71.40%	
BMI	25.7 ± 5.8	26.4 ± 3.4	24.8 ± 1.8	na	0.611
Suicide attempters	33.3%	16.7%	16.7%	–	–
Elevated albumin ratio	–	66.7%	50.0%	–	–
MRM cohort					
Group size	40	11	13	27	–
Subgroups	Recurrent depressive disorder (30)	Current episode mixed (4)	Paranoid (7)	–	–
		Current episode manic (3)			
	Depressive episode (10)	Current episode depression (4)	Undifferentiated (6)		
Age	48 ± 11.3	46.6 ± 16.5	40.4 ± 14.8	49.2 ± 9.6	0.0932
	(20–69)	(19–74)	(23.8–78.2)	(28.2–65.6)	
Sex F/M	25/15	4/7	6/7	17/10	–
Female	62.5%	36.4%	46.2%	63.0%	
BMI	25.4 ± 4.4	25.1 ± 4	26.4 ± 3.6	na	0.6697
Suicide attempters	27.5%	27.3%	15.4%	–	–
Elevated albumin ratio	–	45.5%	38.5%	–	–

Given values for age are mean ± SD and ranges in brackets, *p* values are calculated using Kruskal–Wallis test.

*na* not available, *iTRAQ* isobaric tags for relative and absolute quantitation, *MRM* multiple reaction monitoring.

### Discovery proteomic analysis of CSF obtained from psychiatric patients focusing on MDD

The deep discovery proteomic analysis of 38 CSF samples identified 1795 protein groups (Supplementary Table 6). The analysis quantified 1115 protein groups per CSF sample on average, and the number of quantified protein groups was consistent across the studied groups.

**Fig. 1:**
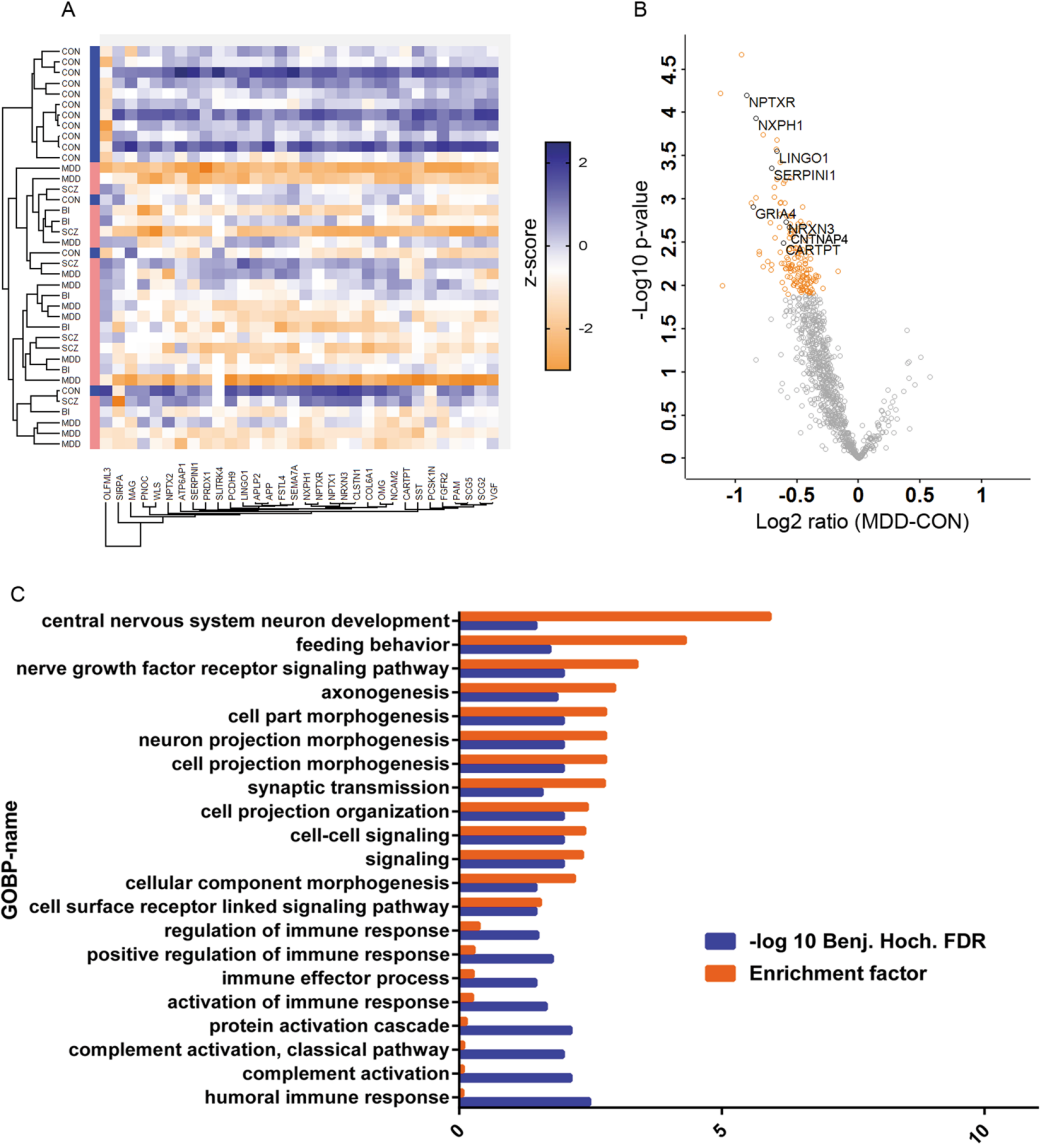
Discovery proteomic analysis of cerebrospinal fluid from psychiatric patients. **a** Hierarchical clustering analysis of differentially regulated proteins in CSF of psychiatric patients demonstrating a clear diverging of the psychiatric patients from the controls. **b** Volcano plot showing differentially regulated protein in CSF of MDD patients vs controls (in orange significant hits). For **a** and **b**, Student’s *t* test with permutation-based FDR (FDR 0.05, 250 randomizations). Shown are gene names. **c** Gene ontology enrichment analysis of differentially regulated proteins in the CSF of MDD patients. Fisher exact test (Benjamini–Hochberg FDR 0.05). GOBP gene ontology biological process.

The comparison of psychiatric disorders (PSY) as a single cumulated group to the controls (PSY vs Con) revealed 32 proteins that were differentially regulated in CSF (Student’s *t* test, permutation-based FDR < 0.05). These proteins were involved in, among other biological functions, myelination (e.g., myelin-associated glycoprotein), synaptic transmission (e.g., NRXN3), Wnt signaling (Wnt ligand secretion mediator), and somatostatin signaling. Non-supervised hierarchical clustering analysis of the studied samples using the differentially regulated proteins demonstrated a clear divergence of the psychiatric patients from the Con group (Fig. 1a).

The comparison of the CSF proteome obtained from MDD patients and controls (MDD vs Con) identified 161 downregulated proteins after correction for multiple testing (Student’s *t* test, permutation-based FDR < 0.05; Supplementary Table 7). The downregulated proteins included several synaptic proteins such as NRXN3, NXPH1, and CNTNAP4 (Fig. 1b). The GO term enrichment analysis associated the differentially regulated proteins with 21 BPs including axonogenesis, synaptic transmission, and regulation of immune response (Fisher test, Benjamini–Hochberg FDR < 0.05; Fig. 1c). Furthermore, the analysis associated the differentially regulated proteins with the following cellular compartments: extracellular region, intrinsic to membrane, and blood microparticle (Fisher exact test, Benjamini–Hochberg FDR < 0.05).

**Fig. 2:**
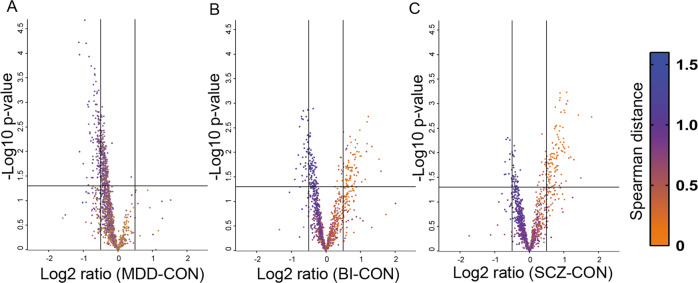
Impaired blood–CSF barrier function in schizophrenia and bipolar patients. **a** Volcano plot of differentially regulated proteins in CSF of MDD patients in comparison to the controls. **b** Volcano plot of differentially regulated proteins in CSF of BI patients in comparison to the controls. **c** Volcano plot of differentially regulated proteins in CSF of SCZ patients in comparison to the controls. The coloring scale is according to Spearman distance with albumin CSF levels in the samples (albumin profile). The horizontal line at –log 10 *p* value that equals 1.3 refers to the significance level. Vertical lines at log2 ratio fold change equal to 0.5 and −0.5.

The comparisons BI vs Con, SCZ vs Con, BI vs MDD, and SCZ vs BI identified changes in the levels of 197 (116 decreased and 81 increased), 153 (47 decreased and 106 increased), 99 (increased), and 9 (2 decreased and 7 increased) proteins, respectively (Student’s *t* test, *p* < 0.05; Supplementary Table 7). However, these proteins did not reach the level of significance after correction for multiple testing using a permutation-based FDR cut-off 0.05. The comparison of SCZ and depression CSF proteomes (SCZ vs MDD) demonstrated 146 upregulated proteins (Student’s *t* test, *p* < 0.05; Supplementary Table 7) and secreted frizzled related protein 4 after correction for multiple testing (Student’s *t* test, permutation-based FDR < 0.05). The majority of the upregulated proteins in SCZ and BI patients in comparison to the controls demonstrated similarity to CSF albumin profile (Fig. 2).

### Validation of the proteomic changes in a larger cohort of MDD patients

To validate the proteomic changes identified in the discovery experiment, we developed a multiplex assay using the targeted MS proteomic approach (MRM) for a group of 12 proteins. We specifically measured this panel of proteins in a larger sample cohort. The MRM cohort included the samples that showed changes in the discovery analysis with additional new samples. The MRM cohort consisted of 40 MDD, 13 SCZ, 11 BI, and 27 controls. Our developed MRM assay could quantify 17 proteotypic peptides representing the 12 selected proteins. The intra-assay variation for the peptides was calculated by measuring 4 technical replicates of a control CSF sample, and it was <15% for all peptides (Supplementary Fig. 1). The inter-assay variation for the peptides was calculated by measuring five replicates of a CSF pool (at the beginning and after every 10–12 samples to the end of the run) in each run, and it was <22% for all peptides (Supplementary Fig. 2).

Our protein panel included neurosecretory protein VGF (VGF), LINGO1, CNTNAP4, NRXN3, NXPH1, PCSK1N, SERPINI1, CARTPT, GRIA4, NPTXR, and CBLN4, which all showed significant downregulation in the CSF of MDD in comparison to the controls in the discovery approach (Student’s *t* test, permutation-based FDR < 0.05). In addition, our protein panel included CNDP1 that was significantly downregulated in SCZ patients in comparison to the controls in the discovery experiment (Student’s *t* test, *p* value <0.05). We selected these particular proteins from the proteins that showed differential expression in the discovery analysis based on a literature analysis focusing on the following points: enrichment of the protein expression in the brain, function of the protein, and/or a previous description of a possible association with a psychiatric disorder.

**Fig. 3:**
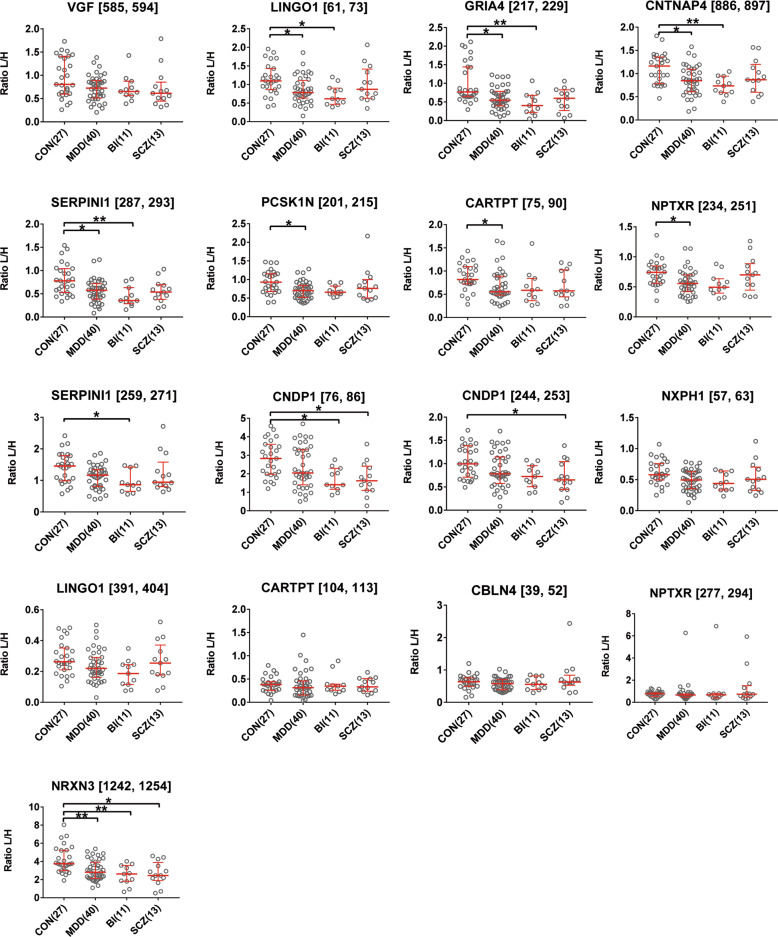
Validation analysis of differentially regulated proteins (peptides) in cerebrospinal fluid of psychiatric disorders. Shown are median with an interquartile range of the ratios of light peptides to spiked synthetic heavy peptides in the different patient cohorts (number of patients in brackets). Asterisks refer to statistically significant differences with Kruskal–Wallis test and Dunn’s Multiple comparison test, **p < 0.01, *p < 0.05. CON controls, MDD major depressive disorder, BI bipolar disorder, SCZ schizophrenia, VGF neurosecretory protein VGF, LINGO1 leucine-rich repeat and immunoglobulin-like domain-containing nogo receptor-interacting protein 1, GRIA4 glutamate ionotropic receptor AMPA type subunit 4, CNTNAP4 contactin-associated protein-like 4, SERPINI1 neuroserpin, PCSK1N proprotein convertase subtilisin/kexin type 1 inhibitor, CARTPT cocaine- and amphetamine-regulated transcript protein, NPTXR neuronal pentraxin receptor, CNDP1 carnosine dipeptidase 1, NXPH1 neurexophilin 1, CBLN4 cerebellin-4, NRXN3 neurexin 3.

The MRM measurements confirmed the discovery data on 9 out of the 12 measured proteins. In line with the findings of the discovery analysis, the VGF [585–594] peptide showed a trend to downregulation in all psychiatric disorders in comparison to the control group in the MRM cohort. MDD and BI disorder patients showed significant downregulation in the CSF levels of LINGO1 [61–73] (*p* < 0.05, *p* < 0.05), GRIA4 [217–229] (*p* < 0.05, *p* < 0.01), CNTNAP4 [886–897] (*p* < 0.05, *p* < 0.01), and SERPINI1 [287–293] (*p* < 0.05, *p* < 0.01) in comparison to the controls. MDD patients showed significant downregulation in the CSF levels of PCSK1N [201–215] (*p* < 0.05), NPTXR [234–251] (*p* < 0.05), and CARTPT [75–90] (*p* < 0.05) in comparison to the controls. SERPINI1 [259–271] was significantly downregulated in the CSF of BI patients (*p* < 0.05) in comparison to the controls. CNDP1 [76–86] was significantly downregulated in the CSF of BI (*p* < 0.05) and SCZ (*p* < 0.05) patients in comparison to the controls, and CNDP1 [244–253] was significantly downregulated in the CSF of SCZ patients (*p* < 0.05) in comparison to the controls. NXPH1 [57–63] and LINGO1 [391-404] showed a trend to downregulation in MDD patients in comparison to the controls. CARTPT [104–113], CBLN4 [38–49, 51, 52], and NPTXR [277–294] CSF levels did not differ between the studied groups of psychiatric disorders and the control group in the MRM cohort. NRXN3 [1242–1254] was significantly downregulated in the CSF of all studied psychiatric disorder groups (MDD: *p* < 0.01, BI: *p* < 0.01, SCZ: *p* < 0.05) in comparison to the control group (Fig. 3).

**Fig. 4:**
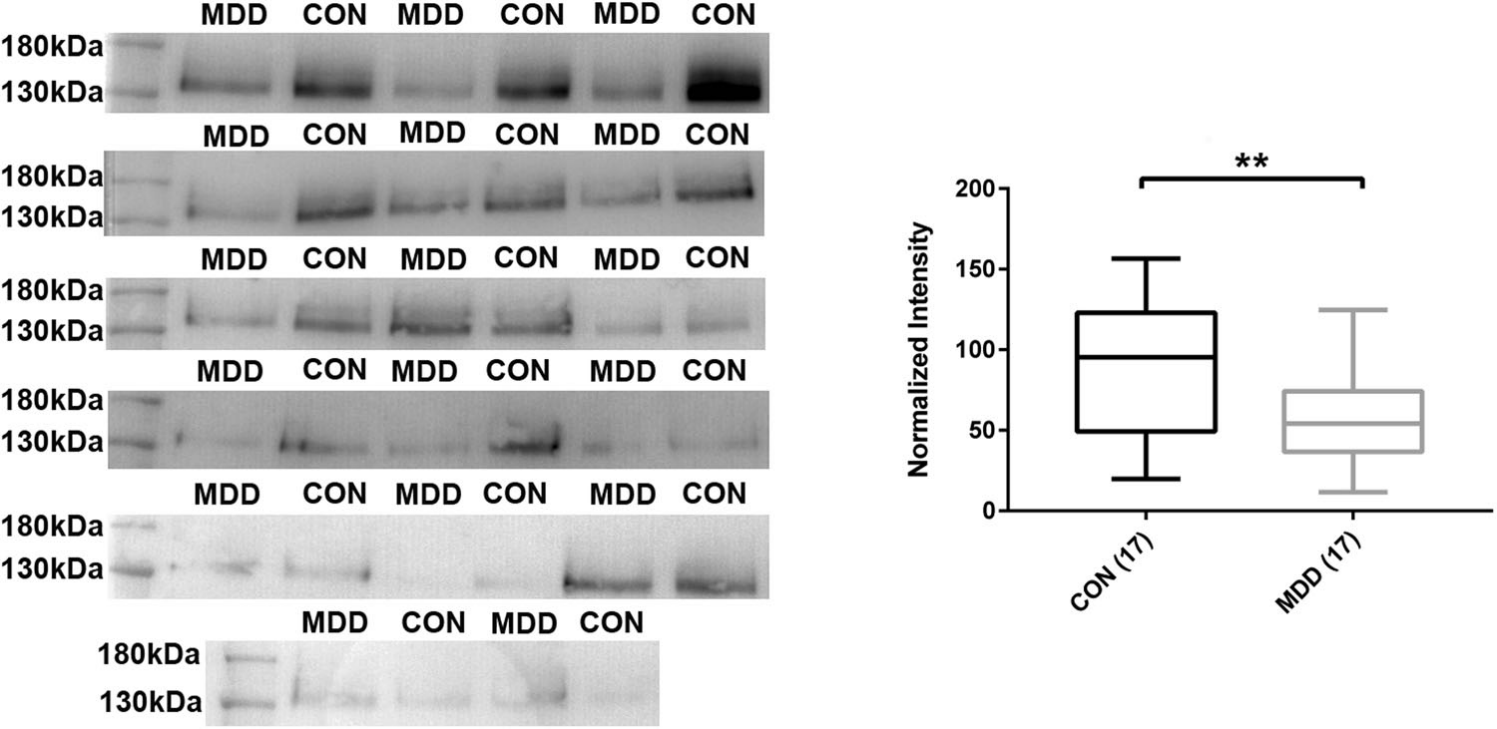
Decreased neurexin 3 levels in cerebrospinal fluid of major depressive disorder. Boxes are the median concentrations and interquartile range; whiskers are minimum and maximum. Asterisks refer to statistically significant differences with *T* test, **p < 0.01. CON: 17 controls and MDD: 17 major depressive disorder. The patients and controls were selected randomly from the validation cohort.

In addition, in line with the discovery and targeted proteomic findings, a downregulation of NRXN3 levels in the CSF of psychiatric disorders in comparison to the controls was detected using western blot (Fig. 4).

To assess the gender’s effect on the levels of measured peptides in CSF, we performed a two-way ANOVA test on the MDD and controls data, we set the disease as a first variable and the gender as a second variable, and the analysis pointed out a weak gender effect on the CSF levels of CARTPT [104–113] (interaction *p* = 0.0393), NXPH1 [57–63] (interaction *p* = 0.0174), VGF [585–594] (interaction *p* = 0.0343), and CNDP1 [244–253] (interaction *p* = 0.0215), while the analysis showed no gender’s effect on the CSF levels of all other measured peptides. Furthermore, the analysis demonstrated a pronounced disease’s effect on the CSF levels of the following peptides: VGF [585–594] (*p* < 0.05), LINGO1 [61–73] (*p* < 0.05), GRIA4 [217–229] (*p* < 0.001), CNTNAP4 [886–897] (*p* < 0.01), SERPINI1 [287–293] (*p* < 0.01), PCSK1N [201–215] (*p* < 0.01), NPTXR [234–251] (*p* < 0.05), CARTPT [75–90] (*p* < 0.05), SERPINI1 [259–271] (*p* < 0.05), and NRXN3 [1242–1254] (*p* < 0.01). The analysis showed no significant differences in the CSF levels of all measured peptides between male and female participants (*p* > 0.05; Supplementary Fig. 3).

The CSF levels of the measured peptides did not correlate with age and CSF/serum albumin ratio for the controls (Supplementary Table 8). We observed a weak correlation between NXPH1 [57, 63] (*r* = 0.52, *p* = 0.013), CNTNAP4 [889, 897] (*r* = 0.42, *p* = 0.049) and MADRS in 22 depression patients from whom this assessment scale was available. The CSF levels of the other studied peptides did not correlate with MADRS (Supplementary Table 8). To investigate the medication’s effect, we re-distributed 36 of our MDD patients according to their treatment protocols into the following subgroups (17 receiving antidepressants, 5 receiving double therapy of antidepressants and benzodiazepine, 10 double therapy of antidepressants and antipsychotics, and 4 receiving triple therapy of antidepressants, benzodiazepine, and antipsychotics). We observed no obvious differences between different MDD patients on different treatment protocols and decreased levels of LINGO1 [61–73], GRIA4 [217–229], CNTNAP4 [886–897], SERPINI1 [287–293], PCSK1N [201–215], NPTXR [234–251], CARTPT [75–90], and NRXN3 [1242–1254] in all MDD subgroups in comparison to the controls (Supplementary Fig. 4).

For further validation, we regrouped the MDD and control samples of the MRM cohort into two smaller separated cohorts. The first cohort included the 12 MDD vs 14 controls samples that showed changes in the discovery iTRAQ analysis (iTRAQ-MDD vs iTRAQ-Con) and the second cohort included the added validation 28 MDD vs 13 controls samples (new-MDD vs new-Con). We checked for the CSF levels of the eight proteins that showed differential expression in MDD in the MRM cohort in these two cohorts independently. In line with the results of the combined MRM cohort, the independent analysis of these two cohorts revealed four proteins (CNTNAP4, SERPINI1, NRXN3, and GRIA4) that were significantly downregulated in the CSF of MDD patients in both cohorts (iTRAQ-MDD vs iTRAQ-Con, *p* < 0.05 and new-MDD vs new-Con, *p* < 0.05). For the other four proteins (PCSK1N, LINGO1, CARTPT, and NPTXR), we observed a downregulation in both cohorts. The difference was significant in the discovery iTRAQ 12 MDD vs 14 controls samples but it did not reach the level of significance (*p* < 0.05) in the other cohort (Supplementary Fig. 5).

## Discussion

The study presents the first deep proteomic investigation on the CSF of MDD, BI disorder, and SCZ. Overall, we identified 1795 protein groups and 12,667 peptides. To our knowledge, this is the most extensive data on the CSF proteome focusing on psychiatric diseases, to date.

The study provides evidence on a possible synaptic dysfunction in MDD at the proteomic level. There is increasing size of evidence suggesting synaptic dysfunction and impaired integrity of certain brain circuits as molecular mechanisms underpinning MDD^25,26^. Previous imaging studies have reported decreased volume and functional activity of limbic brain regions in MDD^27,28^. Functional imaging studies demonstrated altered connectomics between several brain regions in MDD^29^. The studies of postmortem MDD brains demonstrated decreased expression of synaptic-related genes^30,31^ and alteration of phosphorylation of synaptic proteins^32^. Consistently, animal studies on rodents demonstrated reduced synaptic density upon stress^33^. On the genetic level, MDD brains showed decreased expression of some synaptic genes^30^. Fast-acting antidepressants, particularly ketamine, associated with induction of synaptogenesis. Our discovery and targeted proteomic analysis reveal decreased CSF levels of several synaptic proteins in MDD patients in comparison to the controls. In addition, the GO term analysis associated the differentially regulated proteins in MDD with the synaptic transmission. Putting all together, the altered CSF levels of the reported synaptic proteins refer to synaptic dysfunction in MDD.

Our study presents the first report on significantly decreased levels of NRXN3 and CNTNAP4 in the CSF of major psychiatric disorders, mainly MDD. NRXN3 belongs to the family of neurexin proteins that are synaptic cell adhesion molecules playing a crucial role in regulating synaptic properties and trans-synaptic signaling^34^. Mutations and polymorphisms of the NRXN3 gene have been associated with autism^35^, addiction behavior^36,37^, and SCZ^38^. A previous study demonstrated a distinct role of NRXN3 in the regulation of postsynaptic AMPA receptors as well as the release of GABA in the synapses of different brain regions^39,40^. Recent studies reported the important role of the presynaptic CNTNAP4 in the regulation of GABAergic and dopaminergic synaptic transmission^41^. In line with previously published reports, our proteomic analysis demonstrated the feasibility of measuring synaptic proteins, e.g., NRXN3 in CSF^42^. Synapses have a dynamic nature, there may be a continuous turnover of the synaptic proteins^43^, and it is not completely understood how synaptic proteins can end up in CSF. However, several mechanisms including dendritic exocytosis^44^, diffusion in brain extracellular space^45^, secretion^46^ and extracellular vesicles^47^ may play a role in the transmission of these proteins to CSF. Given the importance of NRXN3 and CNTNAP4 in mediating trans-synaptic properties as well as synaptic transmission, our data regarding decreased levels of NRXN3 and CNTNAP4 in CSF of psychiatric patients, mainly MDD in comparison to the controls, indicates impaired synaptic signaling in MDD.

Considering CSF/serum albumin ratios is of relevance in CSF biomarker studies. In line with previously published data^48,49^, we observed increasing blood–CSF barrier permeability in SCZ and BI patients (Table 1). Despite the difference in the percentage of patients with elevated CSF/serum albumin ratio in the studied groups, the analysis demonstrates significant downregulation of LINGO1 [61–73], GRIA4 [217–229], CNTNAP4 [886–897] and SERPINI1 [287–293] in both MDD and BI patients in comparison to controls, respectively, and NRXN3 in MDD, BI, and SCZ in comparison to the controls, respectively (Fig. 4). In addition, no correlation was detected between the levels of our reported proteins and the CSF/serum albumin ratios. This suggests a limited effect of blood–CSF barrier function on the levels of these proteins.

In addition, we observed that the majority of the upregulated proteins in SCZ and BI patients in comparison to the controls demonstrated similarity to the CSF albumin profile. Albumin spills into CSF from blood, and the similarity of a protein profile to the albumin profile may indicate that this protein is mainly blood derived. Elevation in the levels of the proteins showing similar profiles to albumin refers to an impaired CSF–blood barrier function and this in line with the detected elevated albumin quotient in SCZ and BI patients.

Major psychiatric disorders share some symptoms and they may share some pathophysiological mechanisms as reported in a large study of psychiatric brains at the transcriptional level^50^. We identified shared changes in CSF levels of some proteins across two or three major psychiatric disorders including MDD, BI disorder, and SCZ (e.g., NRXN3, CNDP1, LINGO1) indicating shared neuropathology across these diseases. Other proteins like PCSK1N [201–215], NPTXR [234–251], and CARTPT [75–90] showed specific downregulation in MDD and may present interesting biomarkers for differential diagnosis. However, the small number of SCZ and BI patients included in the study limits the power of this finding. Further validation studies on the utility of the reported biosignatures for differential diagnosis in a larger patient cohort would be highly desirable, although the levels of the reported proteins were comparable and shown to be decreased in MDD patients on different treatment protocols in comparison to the controls. It is of great interest to investigate the CSF levels of the reported proteins in drug-naive MDD patients in future studies to elaborate on the antidepressant’s effect on the levels of these proteins in CSF.

The developed MRM method in our study included a single proteotypic peptide per protein for some of our investigated candidates (CBLN4, PCSK1N, CNTNAP4, GRIA4, NRXN3, NXPH1, and VGF), and this would be a potential for improvement in a future approach. It would be interesting to characterize the reported proteomic changes more thoroughly using further peptides and clarify any possible differences in the levels of other peptides of the reported proteins.

In summary, the study presents the first deep proteomic investigation on the CSF of MDD, BI disorder, and SCZ. The depth obtained in this analysis highlights the dynamic nature of the CSF proteome. It emphasizes that CSF presents a rich resource for biomarker discovery and pathophysiology studies in psychiatric disorders. It reveals a biosignature of decreased synaptic protein levels including NRXN3 and CNTNAP4 in the CSF of psychiatric disorders, mainly MDD. It presents a novel method to measure 12 brain-enriched proteins simultaneously with high specificity in CSF. We anticipate that the acquired dataset is of great interest to provide deeper insights into the underpinning pathophysiology of psychiatric disorders, mainly MDD, and demonstrate proteins that constitute a biosignature of MDD for further validation studies.

## Supplementary information

Supplementary-Information

Supplementary-Table-7

Supplementary-Table-5

Supplementary-Table-6
