# Supplementary material for: Proteomic analysis reveals a biosignature of decreased synaptic protein in cerebrospinal fluid of major depressive disorder
Source: Transl Psychiatry. 2020 May 12;10:144. doi: 10.1038/s41398-020-0825-7 (PMC7217933; doi:10.1038/s41398-020-0825-7)
Supplement: Supplementary file 1 — Supplementary-Information [file 41398_2020_825_MOESM1_ESM.docx]

Supplementary Table 1. Sequences of the standard heavy peptides.

| **Synthetic Heavy Peptide Name** | **Sequence** | **Stable Isotope-Labeled Residue** |
| --- | --- | --- |
| CBLN4 [37-55] | EGKCLVVCDSNPATDS(K)GS | Lysine (K), +8Da |
| PCSK1N [199-218] | LGRILAGSADSEGVAAP(R)RL | Arginine (R), +10Da |
| VGF QPrEST | PAPAPA(R)DEL PDWNEVLPPW D(R)EEDEVYPP GPYHPFPNYI (R)P(R)TLQPPSA L(R)(R)(R)HYHHAL PPS(R)HYPG(R)E AQA(R)(R)AQEEA EAEE(R)(R)LQEQ EELENYIEHV | Arginine (R), +10Da oer residue |
| CARTPT [102 -116] | CPRGTSCNSFLL(K)CL | Lysine (K), +8Da |
| CARTPT [73-93] | EKKYGQVPMCDAGEQCAV(R)KG | Arginine (R), +10Da |
| CNDP1 [242-256] | GTRGNSYFMVEV(K)CR | Lysine (K), +8Da |
| CNDP1 [74-89] | LFRMMAV AADTLQ(R)LG | Arginine (R), +10Da |
| CNTNAP4 [884-900] | NMKEASLQVDQLTP(K)TQ | Lysine (K), +8Da |
| GRIA4 [215- 232] | IERLQNILEQIVSVG(K)HV | Lysine (K), +8Da |
| LINGO1 [389-407] | FNRQQPTCATPEFVQG(K)EF | Lysine (K), +8Da |
| LINGO1 [59-76] | RKRFVAVPEGIPTET(R)LL | Arginine (R), +10Da |
| NPTXR [234-253] | SKMDQLEGQLLAQVLALE(K)ER | Lysine (K), +8Da |
| NPTXR [277-297] | GRVAELEHGSSAYSPPDAF(K)IS | Lysine (K), +8Da |
| NRXN3 [1240-1257] | GLKVLNMAAENNPNI(K)IN | Lysine (K), +8Da |
| NXPH1 [55- 66] | ISRLLSQTF(R)GK | Arginine (R), +10Da |
| SERPINI1 [257- 274] | LSRQEVPLATLEPLV(K)AQ | Lysine (K), +8Da |
| SERPINI1 [285- 296] | KQKVEVYLP(R)FT | Arginine (R), +10Da |

Cerebellin-4 (CBLN4), Proprotein Convertase Subtilisin/Kexin Type 1 Inhibitor (PCSK1N), Neurosecretory protein VGF (VGF), Cocaine- And Amphetamine-Regulated Transcript Protein (CARTPT), Carnosine Dipeptidase 1 (CNDP1), Contactin-associated protein-like 4 (CNTNAP4), Glutamate Ionotropic Receptor AMPA Type Subunit 4 (GRIA4), Leucine-rich repeat and immunoglobulin-like domain-containing nogo receptor-interacting protein 1 (LINGO1), Neuronal Pentraxin Receptor (NPTXR), Neurexin 3 (NRXN3), Neurexophilin 1 (NXPH1) and Neuroserpin (SERPINI1).

Supplementary Table 2. Diagnosis of the control group.

| **Control Diagnosis (size)** | **Age (y)** | **Gender** | **NFL pg/ml** |
| --- | --- | --- | --- |
| Numb feeling in the hand | 29.4 | M | 625 |
| Benign paroxysmal positional vertigo (3) | 65.6 | M | 755 |
|  | 54.1 | F | 525 |
|  | 47.4 | M | 425 |
| Migraine (5) | 54.9 | F | 527 |
|  | 46.6 | F | 397.5 |
|  | 47.4 | F | 505 |
|  | 45.7 | F | 865 |
|  | 54.3 | F | 832.5 |
| Anterior ischemic optic neuropathy, DM, arterial hypertension | 64.3 | F | 440 |
| A tension headache (2) | 58.1 | M | 427.5 |
|  | 64.7 | F | 672.5 |
| Retinal ischemia right eye, DM | 48.1 | F | 2347.5 |
| Nonsystematic vertigo, arterial hypertension | 44.7 | F | 575 |
| Radiculopathy, spinal stenosis (3) | 38.0 | M | 387.5 |
|  | 56.2 | F | 662.5 |
|  | 50.7 | M | 852.5 |
| Eyelid Ptosis with swelling of the eyelid may be due to allergic reason, Hypothyroidism | 49.4 | M | 595 |
| Foreign body in the conjunctival sac | 36.0 | M | 487.5 |
| Chronic fatigue syndrome, idiopathic | 45.4 | F | 425 |
| Vasovagal presyncope | 49.4 | F | 1287.5 |
| Labyrinthitis (2) | 46.8 | F | 522.5 |
|  | 62.1 | M | 670 |
| Morbus Menière | 47.7 | F | 482.5 |
| Exclude cerebral ischemia (1) | 50.4 | F | 490 |
| Artery occlusion A. temporalis superior- Eye thrombosis | 41.6 | F | 670 |
| Liability to Pressure Palsies (Plexus brachialis) | 28.2 | M | 587.5 |

Supplementary Table 3. Medications of the MDD, SCZ and BI patients taken at the time of the CSF collection.

| **Diagnose** | **NDRI** | **NaSSA** | **SSRI** | **TCA** | **BZD** | **SSNRIs** | **Non BZD hypnotics** | **Antipsychotic Neuroleptika** | **Lithium** | **Valproate** |
| --- | --- | --- | --- | --- | --- | --- | --- | --- | --- | --- |
| MDD-1 |  |  | Fluoxetine | Trimipramine |  |  |  |  |  |  |
| MDD-2 |  |  | Sertralin | Trimipramine |  |  |  |  |  |  |
| MDD-3 | Bupropion | Mirtazapin |  |  |  |  |  |  |  |  |
| MDD-4 |  |  | Sertralin |  |  |  |  |  |  |  |
| MDD-5 |  |  | Fluoxetine | Trimipramine |  |  |  |  |  |  |
| MDD-6 |  |  | Escitalopram |  |  |  |  |  |  |  |
| MDD-7 |  |  | Escitalopram |  |  |  |  |  |  |  |
| MDD-8 |  | Mirtazapine | Sertralin |  |  |  |  |  |  |  |
| MDD-9 |  |  |  |  |  | Duloxetine |  |  |  |  |
| MDD-10 |  |  | Escitalopram |  |  |  |  |  |  |  |
| MDD-11 |  |  | Escitalopram |  |  |  |  |  |  |  |
| MDD-12 |  |  | Sertralin | Amitriptylin |  |  |  |  |  |  |
| MDD-13 |  | Mirtazapine |  |  |  |  |  |  |  |  |
| MDD-14 |  |  | Fluoxetine |  |  |  |  |  |  |  |
| MDD-15 |  |  |  |  |  | Venlafaxine |  |  |  |  |
| MDD-16 |  | Mirtazapine | Sertralin |  |  |  |  |  |  |  |
| MDD-17 |  | Mirtazapine | Sertralin |  |  |  |  |  |  |  |
| MDD-18 |  | Mirtazapin | Fluoxetine |  | Lorazepam |  |  |  |  |  |
| MDD-19 |  | Mirtazapine | Sertralin |  | Lorazepam |  |  |  |  |  |
| MDD-20 |  | Mirtazapine | Sertralin |  | Lorazepam |  |  |  |  |  |
| MDD-21 |  | Mirtazapine | Sertralin |  | Lorazepam |  |  |  |  |  |
| MDD-22 |  | Mirtazapine | Sertralin |  | Lorazepam |  |  |  |  |  |
| MDD-23 |  |  | Sertralin |  |  |  |  | Olanzapine |  |  |
| MDD-24 |  |  | Escitalopram |  |  |  |  | Quetiapine |  |  |
| MDD-25 |  |  |  | Trimipramine |  | Venlafaxine |  | Risperidone |  |  |
| MDD-26 |  |  |  |  |  | Duloxetine |  | Quetiapine |  |  |
| MDD-27 |  |  | Sertralin |  |  |  |  | Quetiapine |  |  |
| MDD-28 |  |  | Fluoxetine |  |  |  |  | Quetiapine |  |  |
| MDD-29 |  |  | Sertralin |  |  |  |  | Olanzapine |  |  |
| MDD-30 |  |  | Sertralin |  |  |  |  | Quetiapine |  |  |
| MDD-31 |  |  | Sertralin |  |  |  |  | Olanzapine |  |  |
| MDD-32 |  |  |  |  |  | Duloxetine |  | Quetiapine | lithium carbonate |  |
| MDD-33 |  |  |  |  |  |  |  | Risperidone |  |  |
| MDD-34 |  |  |  |  |  |  |  |  |  |  |
| MDD-35 |  |  | Sertralin |  | Lorazepam |  |  | Quetiapine |  |  |
| MDD-36 |  |  | Sertralin |  | Lorazepam |  | Zopiclone | Quetiapine |  |  |
| MDD-37 |  |  | Sertralin |  | Lorazepam |  |  | Olanzapine |  |  |
| BI-1 |  |  |  |  |  | Venlafaxine |  | Risperidone |  |  |
| BI-2 |  | Mirtazapine | Sertraline |  | Lorazepam | Venlafaxine |  | Quetiapine | Lithium Carbonate |  |
| BI-3 | Bupropion |  |  |  |  |  |  |  | Lithium Carbonate |  |
| BI-4 |  |  | Sertraline |  | Lorazepam |  |  |  | Lithium Carbonate |  |
| BI-5 |  |  |  |  |  |  |  | Aripiprazole+Quetiapine | Lithium Carbonate |  |
| BI-6 |  |  |  |  |  |  |  |  | Lithium Carbonate |  |
| BI-7 |  |  |  |  | Lorazepam |  |  |  | Lithium Carbonate |  |
| BI-8 |  |  |  |  |  |  |  | Quetiapine |  |  |
| BI-9 |  |  |  |  |  |  |  | Risperidone |  |  |
| BI-10 |  |  |  |  | Lorazepam |  |  | Olanzapine+Haloperidol |  |  |
| BI-11 |  |  |  |  | Diazepam |  |  | Haloperidol |  | Valproate |
| SCZ-1 |  |  |  |  |  |  |  | Aripiprazole |  |  |
| SCZ-2 |  |  |  |  | Lorazepam |  |  | Haloperidol |  |  |
| SCZ-3 |  |  |  |  | Lorazepam |  |  | Quetiapine |  |  |
| SCZ-4 |  |  |  |  | Lorazepam |  |  | Amisulpride |  |  |
| SCZ-5 |  |  |  |  |  |  |  | Aripiprazole+Olanzapine |  |  |
| SCZ-6 |  |  |  |  |  |  |  | Risperidone |  |  |
| SCZ-7 |  |  |  |  |  |  |  | Aripiprazole |  |  |
| SCZ-8 |  |  |  |  |  |  |  | Amisulprid+Risperidon |  |  |
| SCZ-9 |  |  |  |  |  |  |  | Aripiprazole |  |  |
| SCZ-10 |  |  |  |  |  |  |  | Clozapine |  |  |
| SCZ-11 |  |  |  |  |  |  |  | Risperidone |  |  |

*The medications of 2 schizophrenia and 3 major depressive disorder patients were not available. CON = controls, MDD = major depressive disorder, BI = bipolar disorder and SCZ = schizophrenia. NDRI: norepinephrine-dopamine reuptake inhibitor,NaSSA: noradrenergic and specific serotonergic antidepressant,SSRI: selective serotonin reuptake inhibitor, TCA: tricyclic antidepressant, SSNRI: serotonin-norepinephrine reuptake inhibitor, BZD: Benzodiazepine.

Supplementary Table 4. The gradient of the developed MRM assay.

| Agilent 1260 (loading pump) | | Eksigent MicroLC200 | |
| --- | --- | --- | --- |
| Flow rate: 200µL/min | | Flow rate: 15µL/min | |
| Time | %B | Time | %B |
| 0 | 1 | 0 | 5 |
| 0.5 | 1 | 0.25 | Column connected |
| 1 | 30 | 10 | Column disconnected |
| 2 | 30 | 10.1 | 30 |
| 2.1 | 30 | 10.3 | 98 |
| 9.5 | 30 | 11.5 | 98 |
| 9.6 | 30 | 11.6 | 5 |
| 10.2 | 30 | 12 | 5 |
| 10.5 | 80 | 12.1 | 98 |
| 14 | 80 | 13.5 | 98 |
| 14.1 | 1 | 13.6 | 5 |
| 15.5 | 1 | 15.5 | 5 |

Supplementary Figure 1- The intra-assay variation for the measured peptides. Neurosecretory protein VGF (VGF), Neuronal Pentraxin Receptor (NPTXR), Neuroserpin (SERPINI1), Leucine-rich repeat and immunoglobulin-like domain-containing nogo receptor-interacting protein 1 (LINGO1), Cocaine- And Amphetamine-Regulated Transcript Protein (CARTPT), Neurexin 3 (NRXN3), Proprotein Convertase Subtilisin/Kexin Type 1 Inhibitor (PCSK1N), Cerebellin-4 (CBLN4), Contactin-associated protein-like 4 (CNTNAP4), Neurexophilin 1 (NXPH1), Glutamate Ionotropic Receptor AMPA Type Subunit 4 (GRIA4), and Carnosine Dipeptidase 1 (CNDP1).

Supplementary Figure-2. The inter-assay variation of L/H ratios of all peptides in the runs, the calculation of CV based on five measurements of a CSF pool (at the beginning and after every 10-12 samples to the end of the run) in each run.

Supplementary Figure 3- The gender effect on the measured peptides. Neurosecretory protein VGF (VGF), Leucine-rich repeat and immunoglobulin-like domain-containing nogo receptor-interacting protein 1 (LINGO1), Cerebellin-4 (CBLN4), Contactin-associated protein-like 4 (CNTNAP4), Neuroserpin (SERPINI1), Cocaine- And Amphetamine-Regulated Transcript Protein (CARTPT), Proprotein Convertase Subtilisin/Kexin Type 1 Inhibitor (PCSK1N), Glutamate Ionotropic Receptor AMPA Type Subunit 4 (GRIA4), Neurexin 3 (NRXN3), Neuronal Pentraxin Receptor (NPTXR), Carnosine Dipeptidase 1 (CNDP1), and Neurexophilin 1 (NXPH1). CON = controls, MDD = major depressive disorder. Two-way ANOVA.

Supplementary table 8- correlation of the measured peptides with age, albumin ratio, and MADRS.

| Parameter | Age | | AlbQ | | MDASR | |
| --- | --- | --- | --- | --- | --- | --- |
| Group | Control (27) | [27] | Control (23) | [27] | MDD (22) | [22] |
| Value-Description | Spearman r | P-Value | Spearman r | P-Value | Spearman r | P-Value |
| VGF [585-594] | 0.1481 | 0.461 | -0.03953 | 0.8579 | 0.08577 | 0.7043 |
| LINGO1 [391-404] | 0.06534 | 0.7461 | -0.2757 | 0.2029 | 0.09492 | 0.6744 |
| CBLN4 [39-52] | 0.135 | 0.5021 | 0.06917 | 0.7538 | 0.2613 | 0.2401 |
| CNTP4 [886-897] | 0.1176 | 0.5592 | -0.2648 | 0.222 | **0.4243** | **0.0491** |
| NEUS [259-271] | -0.005496 | 0.9783 | -0.06719 | 0.7607 | 0.3128 | 0.1564 |
| CART [75-90] | 0.1927 | 0.3356 | -0.03656 | 0.8685 | 0.04975 | 0.826 |
| PCSK1 [201-215] | 0.05679 | 0.7784 | -0.253 | 0.2442 | 0.3905 | 0.0723 |
| PDYN [85-96] | -0.04458 | 0.8253 | -0.2569 | 0.2366 | 0.1561 | 0.4879 |
| LINGO1 [61-73] | 0.1411 | 0.4828 | -0.17 | 0.4382 | 0.1372 | 0.5425 |
| NRXN3A [1242-1254] | -0.02229 | 0.9121 | -0.1472 | 0.5026 | 0.4014 | 0.0641 |
| NPTXR [277-294] | 0.0003053 | 0.9988 | -0.1413 | 0.5201 | 0.3105 | 0.1596 |
| NEUS [287-293] | -0.03756 | 0.8525 | -0.02866 | 0.8967 | 0.1818 | 0.418 |
| CART [104-113] | 0.06229 | 0.7576 | -0.2125 | 0.3304 | 0.05661 | 0.8024 |
| CNDP1 [76-86] | 0.1915 | 0.3388 | -0.01186 | 0.9572 | 0.2184 | 0.3288 |
| CNDP1 [244-253] | 0.2131 | 0.2858 | -0.07806 | 0.7233 | 0.3031 | 0.1704 |
| NPTXR [234-250] | -0.05099 | 0.8006 | -0.2164 | 0.3213 | 0.3414 | 0.12 |
| NXPH1 [57-63] | 0.04916 | 0.8076 | -0.1294 | 0.5561 | **0.5186** | **0.0134** |
| GRIA4 [217-229] | -0.07298 | 0.7175 | -0.1245 | 0.5714 | 0.1727 | 0.4422 |
| PDYN [110-117] | -0.09252 | 0.6462 | -0.1858 | 0.3961 | 0.199 | 0.3747 |

Neurosecretory protein VGF (VGF), Leucine-rich repeat and immunoglobulin-like domain-containing nogo receptor-interacting protein 1 (LINGO1), Cerebellin-4 (CBLN4), Contactin-associated protein-like 4 (CNTNAP4), Neuroserpin (SERPINI1), Cocaine- And Amphetamine-Regulated Transcript Protein (CARTPT), Proprotein Convertase Subtilisin/Kexin Type 1 Inhibitor (PCSK1N), Neurexin 3 (NRXN3), Neuronal Pentraxin Receptor (NPTXR), Carnosine Dipeptidase 1 (CNDP1), Neurexophilin 1 (NXPH1) and Glutamate Ionotropic Receptor AMPA Type Subunit 4 (GRIA4). CON = controls, MDD = major depressive disorder. AlbQ: CSF\Serum albumin ratio, Montgomery-Asberg-Depression-Scale (MADRS)

Supplementary Figure 4-Investigation of the medication´s effect. Neurosecretory protein VGF (VGF), Leucine-rich repeat and immunoglobulin-like domain-containing nogo receptor-interacting protein 1 (LINGO1), Cerebellin-4 (CBLN4), Contactin-associated protein-like 4 (CNTNAP4), Neuroserpin (SERPINI1), Cocaine- And Amphetamine-Regulated Transcript Protein (CARTPT), Proprotein Convertase Subtilisin/Kexin Type 1 Inhibitor (PCSK1N), Neurexin 3 (NRXN3), Neuronal Pentraxin Receptor (NPTXR), Carnosine Dipeptidase 1 (CNDP1), Neurexophilin 1 (NXPH1) and Glutamate Ionotropic Receptor AMPA Type Subunit 4 (GRIA4). CON = controls, MDD = major depressive disorder. AD: antidepressants, BZD: benzodiazepine, and AP: antipsychotic. Asterisks refer to statistically significant differences with unpaired Kruskal-Wallis test and Dunn's post hoc test, **p < 0.01, *p < 0.05. Group A: peptides showed significantly decrease CSF levels in MDD in comparison to the controls. Group B: peptides showed no changes in CSF levels in MDD in comparison to the controls.

Supplementary figure-5. Independent validation of differentially regulated proteins (peptides) in cerebrospinal fluid of major depressive disorder using multiple reaction monitoring (MRM). Shown are median with an interquartile range of the ratios of light peptides to spiked synthetic heavy peptides in the different patient cohorts (number of patients in brackets). Asterisks refer to statistically significant differences with two-tailed Mann-Whitney- test ***p < 0.001, **p < 0.01, *p < 0.05. CON = controls, MDD = major depressive disorder. Leucine-rich repeat and immunoglobulin-like domain-containing nogo receptor-interacting protein 1 (LINGO1), glutamate ionotropic receptor AMPA type subunit 4 (GRIA4), contactin associated protein-like 4 (CNTNAP4), neuroserpin (SERPINI1), proprotein convertase subtilisin/kexin type 1 inhibitor (PCSK1N), cocaine- and amphetamine-regulated transcript protein (CARTPT), neuronal pentraxin receptor (NPTXR) and neurexin 3 (NRXN3).
